# Efficacy of once daily darunavir/ritonavir 800/100 mg in PI/r-experienced HIV-1 infected patients with suppressed HIV-1 replication: the RADAR study

**DOI:** 10.1186/1758-2652-13-S4-P41

**Published:** 2010-11-08

**Authors:** J Ghosn, A Chermak, A Houssaini, G Peytavin, S Lambert-Niclot, N Eychenne, L Slama, A Brunet, C Duvivier, A Simon, AG Marcelin, P Flandre, C Katlama

**Affiliations:** 1 AP-HP, Bicetre University Hospital, Internal Medicine and Infectious Diseases, Le Kremlin Bicetre, France; 2 AP-HP, Pitie-Salpetriere University Hospital, Paris, France; 3 Pierre and Marie Curie University, INSERM U-943, Paris, France; 4 AP-HP, Bichat-Claude Bernard University Hospital, Paris, France; 5 AP-HP, Tenon University Hospital, Paris, France; 6 AP-HP, Bicetre University Hospital, Le Kremlin Bicetre, France;; 7 AP-HP, Necker University Hospital, Paris, France

## Background

Once-daily darunavir/ritonavir 800/100 mg is licensed for first-line treatment and data are available in treatment-experienced patients with no resistance-associated mutations to darunavir. We designed an investigator study to evaluate the switch to once-daily darunavir/ritonavir 800/100 mg in treatment-experienced patients with suppressed HIV-1 replication on a twice-daily ritonavir-boosted protease-inhibitor (bid PI/r) containing regimen, i.e. in a setting where genotypic resistance test cannot be performed.

## Methods

In this open-label, noncomparative, multicenter study, patients on a bid PI/r-containing triple combination, with suppressed viral replication, were switched to once-daily darunavir/r 800/100 mg containing triple combination. The primary endpoint was the proportion of patients with plasma HIV-RNA< 50 cp/ml 24 weeks after the switch. Detailed darunavir pharmacokinetic evaluation was performed at Week 4 (W4) and measurement of HIV-RNA in seminal plasma at baseline and W48 in a subset of patients.

## Results

85 patients were enrolled. All had HIV-RNA<50 cp/ml at screening with a median of 478 CD4/mm3 (range 40-1559) and pre-exposure to a median of 2 PI (1-5). 61 patients were currently on lopinavir/r, 18 on fosamprenavir/r, 4 on saquinavir/r and 2 on indinavir/r. At baseline, 15/16 patients had a seminal HIV-RNA< 100 cp/ml and 125 cp/ml for the remaining one. By intent-to-treat analysis (missing=failure), 78/85 patients (92%, CI95 [83;96]) maintained an HIV-RNA<50 cp/ml at W24. 7 patients experienced protocol-defined treatment failure between baseline and W24: 2 had confirmed viral rebound (88 and 70 cp/ml), 1 discontinued study treatment at W4 for adverse event, 3 withdrew their consent and 1 was lost to follow-up. By on-treatment analysis, 78/80 patients (97%, CI95 [91;99]) maintained an HIV-RNA<50 cp/ml at W24. At W4, the median area under the darunavir plasma concentration-time curve measured in 11 patients was 61 380 ng.h/ml (IQR 25-75% 42 094-97 313), darunavir median trough concentration 1340 ng/ml (907-1830) and darunavir half-life was 12.2 h (8.3-13.7). Tolerability of once-daily darunavir/r 800/100 mg was excellent.

## Conclusion

In PI/r-experienced patients with suppressed viral replication on a bid PI/r-containing regimen, switching to once-daily darunavir/r 800/100 mg containing regimen was able to maintain suppression of viral replication and was safe in this setting where genotypic resistance test could not be performed.

